# Composite hemangioendothelioma: report of a rare neoplasm^☆^

**DOI:** 10.1016/j.abd.2025.501166

**Published:** 2025-08-09

**Authors:** Marilda Aparecida Milanez Morgado de Abreu, Carolina Aparecida de Almeida Ferreira, Gisele Alborghetti Nai

**Affiliations:** Department of Dermatology, Universidade do Oeste Paulista, Presidente Prudente, SP, Brazil

*Dear Editor,*

Hemangioendothelioma is the term used to name a group of heterogeneous vascular neoplasms that show an intermediate behavior between hemangiomas, which are completely benign, and angiosarcomas, which are malignant.^1^, ^2^, ^3^, ^4^ It includes the epithelioid, fusiform, retiform, kaposiform, polymorphic lymph nodal and composite variants.^1^ Most are low-grade vascular neoplasms, being locally aggressive, with a high tendency for local recurrence and low metastatic potential.^3^ Composite hemangioendothelioma (CHE) differs from others by disclosing, in histopathology, the combination of different vascular proliferation patterns, both benign and malignant, in the same lesion.^1^, ^2^, ^3^, ^4^

The present report describes a case of CHE, a rarely reported neoplasm.

The project was approved by the Research Ethics Committee, under number 59637316.6.0000.5515.

A 70-year-old female patient reported the appearance of a tumor in the left inframammary region one year after the excision of a clinically diagnosed hemangioma in the same location, which was growing. She reported that approximately two months before the lesion started to grow more rapidly, bleed, and become extremely painful. She had systemic arterial hypertension, had no other morbidities, and had no other significant personal or family history. Dermatological examination revealed the presence of an ulcerated tumor measuring 10 cm, with raised, violaceous edges, a friable and bleeding surface, and a necrotic background, located in the left inframammary region (Fig. 1). There were no palpable regional lymph nodes, and the rest of the physical examination was unremarkable. Routine laboratory tests were normal. Computed tomography scans of the chest and abdomen showed only an ulcerated lesion in the skin and subcutaneous tissue in the left inframammary region, with intense venous contrast uptake. The histopathological examination of the lesion biopsy was compatible with CHE (Fig. 2). The immunohistochemistry was positive for anti-CD31 and anti-CD34 antibodies, common endothelial markers, and anti-factor VIII and anti-Ki67 (10% positivity), which demonstrate the tumor intermediate malignant potential. The lesion was excised with wide safety margins but recurred two years later, and the patient underwent a new surgical excision. However, due to the involvement of the sternum, costal arches, and pericardium, palliative chemotherapy with paclitaxel was started, but the patient died within six months.

**Fig. 1:**
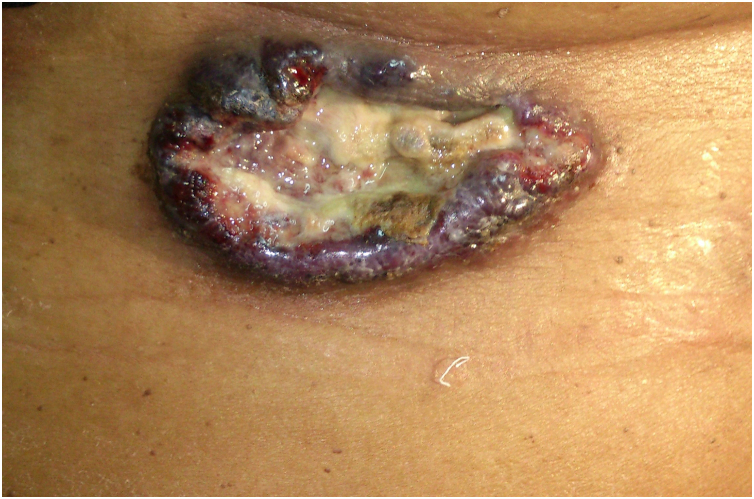
Ulcerated, bleeding tumor with raised, violaceous edges, necrotic background covered by fibrin and purulent secretion, well-defined, located in the left inframammary region. Adjacent skin is normal.

**Fig. 2:**
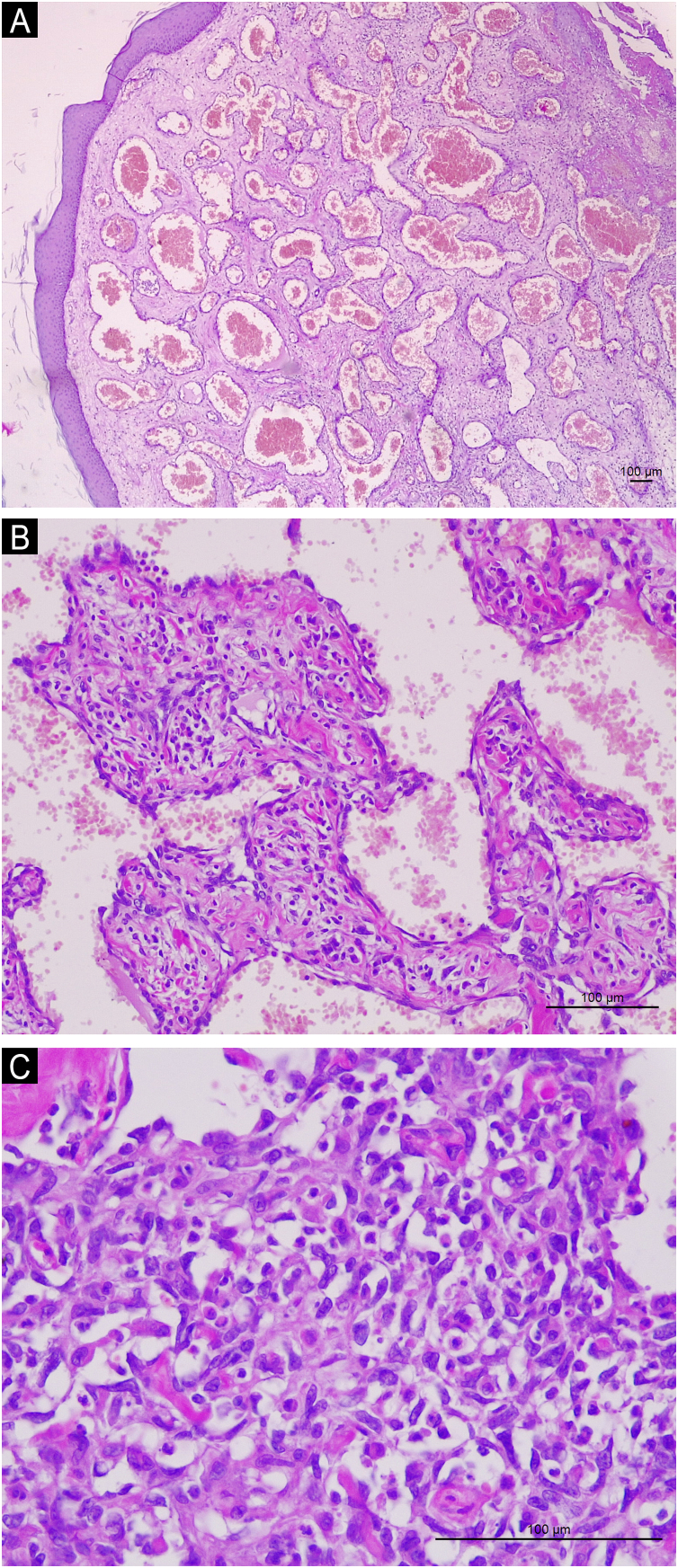
(A) Dilated vascular channels with a cavernous appearance, resembling a common hemangioma in the superficial portion of the biopsy. Magnification of ×40. (B) Observe vessels lined by endothelial cells whose nuclei are often protruding (hobnail), resembling a retiform hemangioendothelioma. Magnification of ×200. (C) Small vascular spaces surrounded by cells with scant cytoplasm and round and fusiform, hyperchromatic and irregular nuclei, suggestive of kaposiform hemangioendothelioma. Magnification of ×400. In (A), (B) and (C): Hematoxylin & eosin, Scale bar: 100 μm.

In 2000, Nayler et al.^5^ reported eight cases of a neoplasm with a variable vascular pattern, showing vascular combinations of benign and malignant neoplasms, naming it CHE,^5^ which was included in the 2002 WHO classification of tumors of soft tissue and bone.^3^

CHE is an extremely rare neoplasm with borderline behavior that differs from other hemangioendotheliomas due to its typical histopathological characteristics.^1^, ^2^, ^3^, ^4^ Its incidence varies by age, with cases reported in children and the elderly,^2^ with a mean age of 39 years, and a predilection for the female sex (2.7:1).^3^ The lesions are clinically indistinguishable from other vascular neoplasms and may manifest as nodules, plaques, or ulcerations, most of which are located at the cutaneous level, mainly in the distal portion of the upper and lower extremities,^1^, ^2^, ^3^, ^4^ with reports of extracutaneous locations.^6^, ^7^, ^8^ Cutaneous locations on the trunk, as in the case described here, are not common.

It is classified as a low-grade malignant neoplasm, rarely evolving with metastases, which occur mainly in the lymph nodes.^2^

Histopathological examination demonstrates an association of benign vascular patterns, such as hemangioma, and malignant ones, such as angiosarcoma, within the same lesion, and the proportion of each component varies.^1^ The histopathological diagnosis requires the presence of at least two variants of hemangioendothelioma.^3^ The predominant components, in most cases, are retiform hemangioendothelioma, epithelioid hemangioendothelioma and spindle cell hemangioma.^1^, ^3^ More rarely, areas of angiosarcoma are also present.

In the present case, the patient reported that the CHE appeared one year after the excision of a hemangioma diagnosed only clinically in the same location, but the histopathological examination was not performed, which leads to the question of whether the lesion was not already a CHE, since the emergence of CHE in a previous scar of another vascular malformation, as well as the malignant transformation of an hemangioma, are extremely rare events.^4^

It is not known what the best therapeutic approach is; there are reports of chemotherapy, interferon alpha, radiotherapy, and excision of the lesion with wide margins.^2^

The authors emphasize the difficulty in diagnosing CHE since the lesion is clinically indistinguishable from other malignant vascular neoplasms and is rare, in addition to requiring the participation of an experienced pathologist.

## Research data availability

Does not apply.

## Scientific Editor-in-Chief

Sílvio Alencar Marque.

## Financial support

None declared.

## Authors’ contributions

Marilda A. M. Morgado de Abreu: Design and planning of the study; critical review of the literature; collection, analysis and interpretation of data; effective participation in research orientation; intellectual participation in the propaedeutic and/or therapeutic conduct of the studied cases; approval of the final version of the manuscript.

Carolina A. de A. Ferreira: Drafting and editing of the manuscript; collection, analysis and interpretation of data; intellectual participation in the propaedeutic and/or therapeutic conduct of the studied cases; critical review of the literature.

Gisele A. Nai: Critical review of the literature; collection, analysis and interpretation of data; effective participation in research orientation; intellectual participation in the propaedeutic and/or therapeutic conduct of the studied cases; approval of the final version of the manuscript.

## Conflicts of interest

None declared.
